# HIV-1 integrase modulates the interaction of the HIV-1 cellular cofactor LEDGF/p75 with chromatin

**DOI:** 10.1186/1742-4690-8-27

**Published:** 2011-04-21

**Authors:** Paulina Astiazaran, Murilo TD Bueno, Elisa Morales, Jeffrey R Kugelman, Jose A Garcia-Rivera, Manuel Llano

**Affiliations:** 1 Department of Biological Sciences. University of Texas at El Paso. 500 West University Ave. El Paso, TX 79968 USA

## Abstract

**Background:**

Chromatin binding plays a central role in the molecular mechanism of LEDGF/p75 in HIV-1 DNA integration. Conflicting results have been reported in regards to the relevance of the LEDGF/p75 chromatin binding element PWWP domain in its HIV-1 cofactor activity.

**Results:**

Here we present evidence that re-expression of a LEDGF/p75 mutant lacking the PWWP domain (ΔPWWP) rescued HIV-1 infection in cells verified to express background levels of endogenous LEDGF/p75 that do not support efficient HIV-1 infection. The HIV-1 cofactor activity of LEDGF/p75 ΔPWWP was similar to that of LEDGF/p75 wild type (WT). A possible molecular explanation for the nonessential role of PWWP domain in the HIV-1 cofactor activity of LEDGF/p75 comes from the fact that coexpression of HIV-1 integrase significantly restored the impaired chromatin binding activity of LEDGF/p75 ΔPWWP. However, integrase failed to promote chromatin binding of a non-chromatin bound LEDGF/p75 mutant that lacks both the PWWP domain and the AT hook motifs (ΔPWWP/AT) and that exhibits negligible HIV-1 cofactor activity. The effect of integrase on the chromatin binding of LEDGF/p75 requires the direct interaction of these two proteins. An HIV-1 integrase mutant, unable to interact with LEDGF/p75, failed to enhance chromatin binding, whereas integrase wild type did not increase the chromatin binding strength of a LEDGF/p75 mutant lacking the integrase binding domain (ΔIBD).

**Conclusions:**

Our data reveal that the PWWP domain of LEDGF/p75 is not essential for its HIV-1 cofactor activity, possibly due to an integrase-mediated increase of the chromatin binding strength of this LEDGF/p75 mutant.

## Background

LEDGF/p75 is a cellular cofactor for HIV-1 DNA integration [1-3] and also participates in the MLL/menin-mediated transcriptional regulation of *Hox *genes [4]. The HIV-1 cofactor activity of LEDGF/p75 requires its simultaneous engagement with the host chromatin and the viral enzyme integrase. LEDGF/p75 mutants that lack their chromatin- or integrase-binding activity are severely defective in their HIV-1 cofactor function [1,2]. Substitution of the chromatin binding domain of LEDGF/p75 by heterologous chromatin binding domains results in proteins that support HIV-1 DNA integration [5-7]. However, the HIV-1 DNA integration site distribution observed in LEDGF/p75-deficient cells expressing these chimeras is altered and determined by the specificity of the added chromatin binding domain [5,6]. These results suggest that the role of the LEDGF/p75 chromatin-binding domain is to provide a tight interaction to the pre-integration complex with the host chromatin.

LEDGF/p75 persists tightly bound to chromatin during all the phases of the cell cycle [8-10]. The chromatin binding activity of LEDGF/p75 is primarily mediated by the functional interaction of the PWWP domain and the AT hook motifs [1,2,7,8,11]. Simultaneous deletion of PWWP domain and AT hook motifs abolished LEDGF/p75 chromatin binding during all the phases of the cellular life cycle [8]. However, deletion of only the AT hook motifs did not alter LEDGF/p75 chromatin binding, while deletion of the PWWP domain decreased the strength of this interaction during interphase and abolished the binding to condensed chromatin during mitosis [7,8,12]. To a markedly lesser extent, the nuclear localization signal and the CR2 and CR4 regions also contribute to the overall binding of LEDGF/p75 to chromatin [11,13].

It is thought that PWWP determines the specificity of the genome-wide location of LEDGF proteins by interacting with chromatin bound proteins [14]. Interaction of the PWWP domain with chromatin seems to be mediated by a solvent-exposed hydrophobic cavity in this domain [15]. Mutation of the conserved residue W21, located in this solvent-exposed hydrophobic cavity, impairs the binding of LEDGF/p75 to chromatin during all phases of the cellular life cycle [15], mimicking the lack of the entire PWWP domain. Mutations of W21 also affect the LEDGF/p75-mediated recruitment of menin/MLL complex to *Hox *genes [4]. Whether or not the PWWP domain of LEDGF/p75 is required for its HIV-1 cofactor activity in the absence of other heterologous chromatin binding domains is still controversial [7,14,15]. Stable re-expression of a LEDGF/p75 ΔPWWP mutant in human LEDGF/p75-deficient CD4+ cells was reported to rescue HIV-1 infection exhibiting approximately 50% of the HIV-1 cofactor activity of LEDGF/p75 WT [7]. However, very low (20.6%) or no HIV-1 cofactor activity (≤0.1%) was observed upon transient expression of LEDGF/p75 ΔPWWP in different LEDGF/p75 *null *mouse fibroblast cell lines [15]. Unexpectedly, in these experiments several LEDGF/p75 PWWP domain point mutants were significantly less active than a LEDGF/p75 mutant lacking the entire PWWP domain [15].

A potential explanation for the discrepancy observed in the HIV-1 cofactor activity of LEDGF/p75 ΔPWWP in human and mouse cells could be that the human LEDGF/p75-deficient cells used in these experiments have regained the ability to express endogenous LEDGF/p75 and this event went unnoticed. These cells were rendered LEDGF/p75-deficient by stable expression of a specific shRNA and were subsequently engineered to stably express LEDGF/p75 ΔPWWP, by transduction with a murine leukemia virus (MLV)-derived viral vector followed by selection, in the presence of G418 [1]. During this process, it is possible that a subpopulation of cells coexpressing endogenous LEDGF/p75 and LEDGF/p75 ΔPWWP was selected. Although expression of endogenous LEDGF/p75 was excluded by immunoblotting analysis [7], LEDGF/p75 levels undetectable by sensitive immunoblots can still mediate HIV-1 DNA integration [1]. This possibility is definitively excluded in the mouse LEDGF/p75 knockout cells.

We have re-evaluated the role of the PWWP domain of LEDGF/p75 in HIV-1 infection using human CD4+ LEDGF/p75-deficient cells. Our data indicate that the PWWP domain of LEDGF/p75 is not essential for its HIV-1 cofactor activity in cells expressing endogenous levels of LEDGF/p75 that do not support HIV-1 infection. In addition, we have found that HIV-1 integrase enhances the chromatin binding activity of LEDGF/p75 ΔPWWP during all the phases of the cell cycle. This effect requires the direct interaction of LEDGF/p75 and HIV-1 integrase. We postulate that the enhancing effect of integrase on the chromatin binding activity of LEDGF/p75 ΔPWWP significantly contributes to the conserved HIV-1 cofactor activity of this mutant.

## Results

### The PWWP domain of LEDGF/p75 is not essential for HIV-1 infection

**Figure 1:**
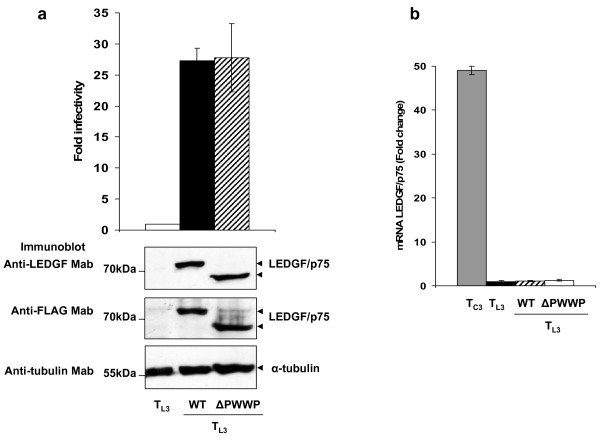
**HIV-1 cofactor activity of LEDGF/p75 ΔPWWP**. (a) T_L3_, and T_L3 _cells expressing LEDGF/p75 WT or LEDGF/p75 ΔPWWP (eleven different cell lines) were challenged with HIVluc and luciferase activity determined five days later. Expression of LEDGF/p75 proteins in these cell lines was documented by immunoblotting with an anti-LEDGF and anti-FLAG Mabs. Errors bars correspond to four independent infection experiments. (b) LEDGF/p75 mRNA levels in T_C3 _and cells used in panel (a). The mRNA levels of endogenous LEDGF/p75 were quantified by real time PCR using specific primers. mRNA levels for LEDGF/p75 were normalized to those of GAPDH in the same samples. Errors bars correspond to triplicate real-time PCR measurements.

In order to evaluate the role of the PWWP domain in the HIV-1 cofactor activity of LEDGF/p75, we engineered human LEDGF/p75-deficient CD4+ T cells, T_L3 _cells, to express C-terminally FLAG tagged LEDGF/p75 ΔPWWP. Expression of the LEDGF/p75 ΔPWWP in these cells was achieved by transduction with an MLV-derived viral vector and verified by immunoblotting (Figure 1a). To further confirm the identity of the re-expressed LEDGF/p75 mutant, genomic DNA was isolated from one T_L3 _LEDGF/p75 ΔPWWP cell line and MLV-derived LEDGF/p75 cDNA was amplified by PCR using primers that hybridize on the MLV genome and on the LEDGF/p75 coding sequence. To avoid detection of non-integrated MLV-delivered LEDGF/p75 ΔPWWP, the analyzed cells were cultured in the selection medium for more than six weeks after MLV-transduction. Using this approach, a DNA fragment of the expected size for the MLV-delivered LEDGF/p75 ΔPWWP cDNA was obtained and its identity verified by DNA sequencing (Data not shown). However, no other DNA fragments indicative of the existence of others MLV-delivered LEDGF/p75 cDNAs in the genome of T_L3 _LEDGF/p75 ΔPWWP cells were observed in these experiments, confirming that the LEDGF/p75 expressed in these cells lack the PWWP domain.

T_L3_, T_L3 _LEDGF/p75 WT and T_L3 _LEDGF/p75 ΔPWWP cells were challenged with a single-round infection HIV-1 luciferase reporter virus (HIVluc) and infectivity was estimated by measuring luciferase five days later. To ensure reproducibility, four independent infections using two different viral doses, one ten fold higher than the other, were considered. A total of eleven independently derived T_L3 _LEDGF/p75 ΔPWWP cell lines were evaluated in these experiments. We observed that stable expression of LEDGF/p75 ΔPWWP in T_L3 _cells rescued HIV-1 infection by 27.8 +/- 5.5 fold (Figure 1a). Similar susceptibility to HIV-1 infection was observed in T_L3 _cells engineered to express LEDGF/p75 WT (26.7 +/- 2.06). No differences in the capability of LEDGF/p75 ΔPWWP to rescue HIV-1 infection in T_L3 _cells were observed when cells were challenged with two different amounts of viruses.

The HIV-1 cofactor activity of LEDGF/p75 ΔPWWP reported here is higher than the previously observed in T_L3 _cells stably expressing this mutant, found to be around 50% of the LEDGF/p75 WT activity [7]. However, both data sets clearly indicate that the PWWP domain is not essential for LEDGF/p75 HIV-1 cofactor activity. These findings in human CD4+ cells notoriously contrast with the required role observed in LEDGF/p75 *null *mouse fibroblasts transiently transfected with LEDGF/p75 ΔPWWP [15]. Interestingly, variability in the HIV-1 cofactor activity of LEDGF/p75 ΔPWWP (≤0.1% to 20.6% of the WT activity) was also observed when different LEDGF/p75 *null *mouse fibroblast cell lines were used [15].

A possible explanation for the observed differences in the HIV-1 cofactor activity of LEDGF/p75 ΔPWWP is the existence of different levels of endogenous LEDGF/p75 in the studied cell lines. LEDGF/p75-deficiency was achieved in T_L3 _cells by stable shRNA mediated-knockdown and therefore during the process of generating T_L3 _LEDGF/p75 ΔPWWP a decrease in the shRNA levels could have occurred, regaining these cells functional levels of endogenous LEDGF/p75. This possibility is excluded in the case of the LEDGF/p75 *null *mouse fibroblasts. In order to evaluate this hypothesis, we determined the levels of endogenous LEDGF/p75 in T_L3_, T_L3 _LEDGF/p75 WT and T_L3 _LEDGF/p75 ΔPWWP cells by real time PCR. In correlation with previous data [1], we observed more than 97% reduction in the endogenous levels of LEDGF/p75 in T_L3 _cells as compared to T_C3 _control cells (Figure 1b). Importantly, more than 97% reduction of endogenous levels of LEDGF/p75 was also observed in both T_L3 _LEDGF/p75 WT and T_L3 _LEDGF/p75 ΔPWWP cells, supporting the notion that the susceptibility of T_L3 _LEDGF/p75 ΔPWWP cells to HIV-1 infection was not determined by the existence of functional amounts of endogenous LEDGF/p75. Therefore, data in figure 1 indicate that the PWWP domain is not essential for the HIV-1 cofactor activity of LEDGF/p75.

### HIV-1 integrase promotes chromatin binding of LEDGF/p75 ΔPWWP

HIV-1 integrase binds to DNA [16,17], and LEDGF/p75 has been reported to increase the DNA binding affinity of HIV-1 integrase in *in vitro *studies [18]. However, it is unknown if integrase has any effect on the chromatin binding activity of LEDGF/p75. Heterologous chromatin binding domains act in *cis *to rescue the HIV-1 cofactor activity of LEDGF/p75 mutants lacking the LEDGF/p75 chromatin binding domain [5-7]. Whether integrase can act in *trans *to promote binding of LEDGF/p75 ΔPWWP to chromatin, resulting in preservation of its HIV-1 cofactor activity, is unknown.

**Figure 2:**
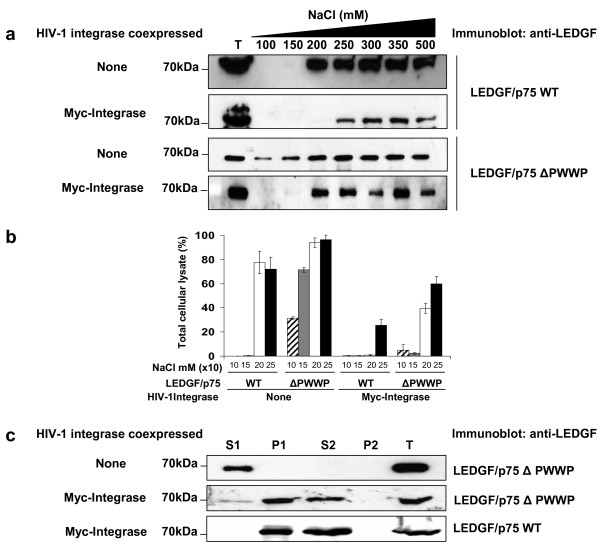
**Effect of HIV-1 integrase on the chromatin binding activity of LEDGF/p75**. (a) The chromatin binding strength of LEDGF/p75 WT and ΔPWWP was determined by the salt extraction method using LEDGF/p75-deficient HEK293T cells stably expressing these LEDGF/p75 proteins alone or together with Myc-tagged HIV-1 integrase. Immunoblots show the amount of LEDGF/p75 extracted from chromatin at different concentrations of NaCl as detected with an anti-LEDGF Mab. T represents a total cellular lysate obtained by boiling the cells in Laemmli buffer. (b) The intensity of different immunoblot bands in panel (a) was quantified by densitometry analysis and expressed as percentage of the intensity of the bands corresponding to the total cellular lysate (T). Errors bars were calculated using two different experiments. (c) Chromatin binding assay. The subcellular distribution of LEDGF/p75 WT or ΔPWWP mutant was evaluated by immunoblotting with an anti-LEDGF Mab in cells stably expressing these LEDGF/p75 proteins alone or together with Myc-tagged HIV-1 integrase. S1 and P2 are non-chromatin bound fractions; P1 and S2 are chromatin-bound fractions, and T is a total cellular lysate. The S1 fraction was obtained by lysing the cells in CSK I buffer containing 150 mM NaCl.

In order to evaluate this hypothesis, we determined the chromatin binding strength of LEDGF/p75 WT and ΔPWWP in LEDGF/p75-deficient cells co-expressing or not co-expressing HIV-1 integrase. Because of the universal character of the cofactor role of LEDGF/p75 in HIV-1 DNA integration (reviewed in [19-21]), we decided to study the effect of HIV-1 integrase on the chromatin binding activity of LEDGF/p75 in LEDGF/p75-deficient HEK293T cells (si1340/1428) [22]. LEDGF/p75 WT or mutants, alone or in combination with HIV-1 integrase, were stably expressed in si1340/1428 cells; and the LEDGF/p75 chromatin binding strength was determined using the salt extraction assay [13]. In the absence of HIV-1 integrase, around 70% of LEDGF/p75 WT was extracted from chromatin at NaCl concentrations above 200 mM. However, this salt concentration extracted less than 1% of LEDGF/p75 WT in cells co-expressing HIV-1 integrase and LEDGF/p75 WT (Figure 2a and 2b). A similar enhancing effect of HIV-1 integrase on the chromatin binding strength of LEDGF/p75 ΔPWWP was observed. In cells expressing LEDGF/p75 ΔPWWP in the absence of HIV-1 integrase, 30% and 70% of LEDGF/p75 ΔPWWP protein was extracted at 100 mM and 150 mM NaCl, respectively (Figure 2a and 2b). However, at these salt concentrations, less than 2% of the LEDGF/p75 ΔPWWP was extracted from cells coexpressing LEDGF/p75 ΔPWWP and HIV-1 integrase (Figure 2a and 2b).

We further evaluated the effect of HIV-1 integrase on LEDGF/p75 ΔPWWP chromatin binding by performing a chromatin binding assay [8]. Using this assay, it has been previously demonstrated that LEDGF/p75 ΔPWWP equally distributes in the non-chromatin bound (S1) and chromatin bound (P1 and S2) fractions [7,8]. In order to obtain a more defined distribution pattern of LEDGF/p75 ΔPWWP among these subcellular fractions, we increased the NaCl concentration of the initial lysis buffer (CSK I buffer) from 100 mM to 150 mM (CSK I-150 buffer). In correlation with the results obtained with the salt extraction assay (Figure 2a and 2b), we found that in the absence of HIV-1 integrase, LEDGF/p75 ΔPWWP was significantly extracted in the S1 fraction when cells were lysed in the CSK I-150 buffer (Figure 2c). However, in cells co-expressing HIV-1 integrase, LEDGF/p75 ΔPWWP was only minimally extracted in the non-chromatin bound fraction (S1), while the majority of protein was detected in the chromatin bound fractions (P1 and S2) (Figure 2c). As expected, LEDGF/p75 WT was present exclusively in the chromatin bound fractions when cells were lysed in the presence of 150 mM NaCl (Figure 2c). In summary, results in figure 2 indicated that HIV-1 integrase increases the binding strength of LEDGF/p75 to chromatin.

### The enhancing effect of integrase on LEDGF/p75 chromatin binding activity requires the direct interaction of these two proteins

In order to evaluate the role of direct protein interaction on the ability of integrase to enhance chromatin binding of LEDGF/p75, we investigated HIV-1 integrase and LEDGF/p75 mutants that fail to reciprocally interact. HIV-1 integrase Q168L mutant [23,24] and a LEDGF/p75 lacking the integrase-binding domain (ΔIBD) were evaluated. In these experiments, FLAG-tagged LEDGF/p75 WT, ΔPWWP, or ΔIBD were transiently coexpressed in HEK293T LEDGF/p75-deficient cells alone or with Myc-eGFP-tagged HIV-1 integrase WT or Q168L mutant and the chromatin binding strength of LEDGF/p75 evaluated as described in Figure 2a.

**Figure 3:**
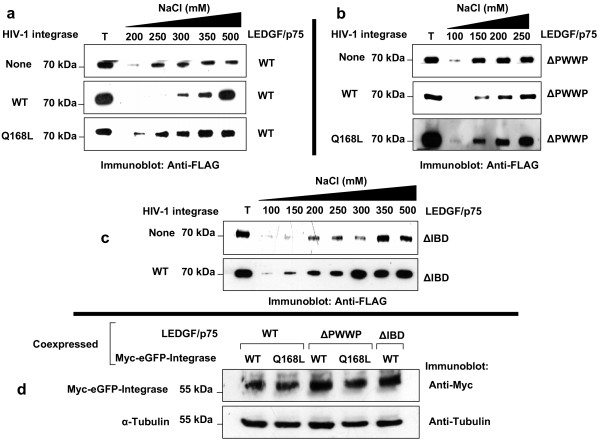
**Effect of HIV-1 integrase Q168L mutant on the chromatin binding activity of LEDGF/p75**. Immunoblots show the effect of transient expression of HIV integrase WT and Q168L mutant on the chromatin binding strength of LEDGF/p75 WT (a) and LEDGF/p75 ΔPWWP (b), and the effect of HIV-1 integrase WT on the chromatin binding strength of LEDGF/p75 ΔIBD (c). LEDGF/p75 was detected with an anti-FLAG Mab. Immunoblots in (d) show the level of expression of HIV integrase WT and Q168L in cells coexpressing LEDGF/p75 WT, LEDGF/p75 ΔPWWP, and LEDGF/p75 ΔIBD analyzed in the experiments represented in (a), (b), and (c), respectively. HIV-1 Integrase was detected with an anti-Myc Mab. T represents a total cellular lysate.

In agreement with our observations in cells stably co-expressing LEDGF/p75 and HIV-1 integrase (Figure 2), transient coexpression of HIV-1 integrase WT increased the chromatin binding activity of LEDGF/p75 WT (Figure 3a) and ΔPWWP (Figure 3b). In the absence of integrase, transiently expressed LEDGF/p75 WT was completely extracted at 250 mM NaCl whereas more than 300 mM NaCl was required to extract LEDGF/p75 WT when HIV-1 integrase WT was coexpressed (Figure 3a). However this effect of integrase was not observed after cotransfection of HIV-1 integrase Q168L and LEDGF/p75 WT. Similarly, LEDGF/p75 ΔPWWP was partially extracted at 100 mM NaCl and required 150 mM NaCl for total extraction in the absence of HIV-1 integrase, whereas when cotransfected with the WT viral protein this LEDGF/p75 mutant was only extracted partially at 150 mM NaCl and required above 200 mM NaCl for complete extraction from chromatin (Figure 3b). On the contrary, co-expression of HIV-1 integrase Q168L mutant with LEDGF/p75 ΔPWWP (Figure 3b) did not increase its chromatin binding activity. The inability of the integrase Q168L mutant to increase the chromatin binding strength of LEDGF/p75 WT or ΔPWWP indicated that the effect of HIV-1 integrase on LEDGF/p75 chromatin binding requires the direct interaction of these two proteins. In further support of this conclusion, we also demonstrated that HIV-1 integrase WT was unable to enhance the chromatin binding activity of a LEDGF/p75 ΔIBD (Figure 3c). In the presence or in the absence of HIV-1 integrase WT, LEDGF/p75 ΔIBD was partially extracted from chromatin at 150 mM NaCl and required more than 200 mM NaCl to be completely extracted (Figure 3c).

The inability of HIV-1 integrase proteins to enhance the chromatin binding activity of LEDGF/p75 proteins in some of the experiments represented in Figure 3a - c was not due to poor expression of the integrase proteins, as demonstrated by anti-Myc immunoblotting (Figure 3d). Data in Figure 3d indicate that similar levels of integrase WT or Q168L were observed in cells analyzed in Figure 3a - c. These results correlated with the similar levels of green fluorescence detected by fluorescence microscopy analysis in the studied cells (not shown).

### HIV-1 integrase allows binding of LEDGF/p75 ΔPWWP to mitotic chromatin

Previous studies have shown that deletion of the PWWP domain in LEDGF/p75 blocks its binding to chromatin during mitosis, indicating that this domain, but not other chromatin binding elements in LEDGF/p75, mediates binding to mitotic condensed chromatin [7,8,12]. In order to evaluate whether HIV-1 integrase can also enhance the binding of LEDGF/p75 ΔPWWP to mitotic chromatin, the subcellular distribution of this mutant was determined in LEDGF/p75-deficient cells coexpressing HIV-1 integrase by immunofluorescence.

**Figure 4:**
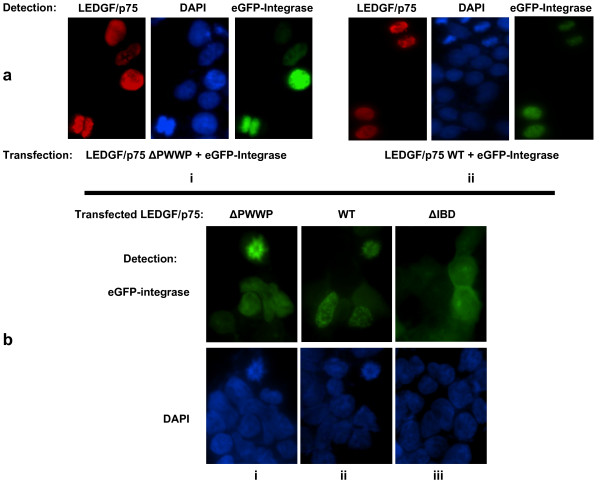
**HIV-1 integrase promotes binding of LEDGF/p75 ΔPWWP to mitotic chromosomes**. (a) Immunofluorescence analysis of LEDGF/p75-deficient cells stably expressing eGFP-tagged HIV-1 integrase and transiently transfected with FLAG-tagged LEDGF/p75 WT (panel ii) or ΔPWWP (panel i). LEDGF/p75 was detected with an anti-FLAG Mab, DAPI was used for detection of chromatin. (b) Integrase-to-chromatin tethering assay. LEDGF/p75-deficient HEK293T cells stably expressing eGFP-tagged integrase were transiently transfected with LEDGF/p75 WT (panel i) or the mutants ΔIBD (panel ii) and ΔPWWP (panel iii) and the subcellular distribution of eGFP-integrase determined by fluorescence microscopy analysis.

Immunostaining of LEDGF/p75-deficient cells coexpressing LEDGF/p75 ΔPWWP and eGFP-tagged HIV-1 integrase indicated that these two proteins colocalized at the nucleus during interphase and on mitotic chromosomes (Figure 4a-i). A comparable distribution was observed in cells coexpressing LEDGF/p75 WT and HIV-1 integrase (Figure 4a-ii). These observations indicated that binding of HIV-1 integrase to LEDGF/p75 ΔPWWP promotes the interaction of this complex with chromatin during mitosis.

Similar results were found using the integrase to chromatin tethering assay [13] where we used LEDGF/p75-deficient cells that stably express eGFP-tagged HIV-1 integrase. In these cells, eGFP-integrase has a pancellular distribution [13]. However, upon transient expression of LEDGF/p75 WT, the viral protein accumulated in the nuclei of cells in interphase and was associated with mitotic chromosomes during cellular division (Figure 4b i). In contrast, expression of LEDGF/p75 ΔIBD did not change the subcellular distribution of eGFP-integrase in these cells (Figure 4b ii). Importantly, transient expression of LEDGF/p75 ΔPWWP caused a change in the subcellular distribution of the eGFP-integrase that was indistinguishable to the one observed upon expression of LEDGF/p75 WT (Figure 4b iii). These evidences demonstrated that HIV-1 integrase promotes interaction of LEDGF/p75 ΔPWWP with mitotic chromosomes.

### HIV-1 integrase failed to promote chromatin binding of a LEDGF/p75 ΔPWWP/AT mutant

**Figure 5:**
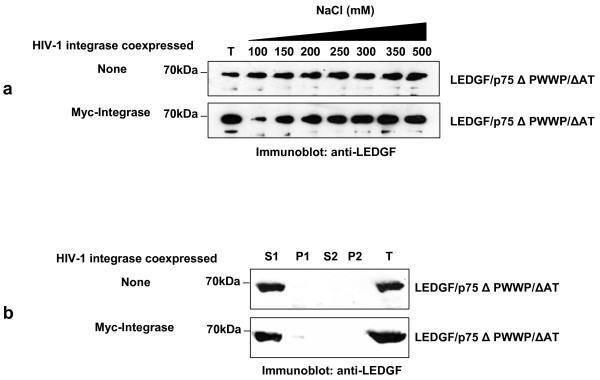
**Effect of HIV-1 integrase on the chromatin binding activity of LEDGF/p75 ΔPWWP/AT**. (a) The chromatin binding strength of LEDGF/p75 ΔPWWP/AT was determined by the salt extraction assay in LEDGF/p75-deficient HEK293T cells stably expressing LEDGF/p75 ΔPWWP/AT alone or together with Myc-tagged HIV-1 integrase. Immunoblots show the amount of LEDGF/p75 ΔPWWP/AT extracted from chromatin at different salt concentrations as detected with an anti-LEDGF Mab. (b) Chromatin binding assay. The subcellular distribution of LEDGF/p75 ΔPWWP/AT was evaluated by cellular fractionation and immunobloting with anti-LEDGF Mab in cells stably expressing this LEDGF/p75 mutant alone or together with Myc-tagged HIV-1 integrase. The S1 fraction was obtained by lysing the cells in CSK I buffer containing 150 mM NaCl. T, S1, P1, S2, and P2 are described in figure legend 2.

Our results suggest that the enhancing effect of integrase on the chromatin binding strength of LEDGF/p75 ΔPWWP could determine the wild type HIV-1 cofactor activity of this mutant. For instance, integrase in the HIV-1 pre-integration complex could enhance in *trans *the chromatin binding of LEDGF/p75 ΔPWWP during viral integration to levels that support full HIV-1 cofactor activity. In this case we should expect that integrase will fail to promote chromatin binding of the chromatin binding defective mutant LEDGF/p75 ΔPWWP/AT since this mutant is also very deficient in HIV-1 cofactor activity [1,2]. Therefore, we explored the effect of integrase on the chromatin binding properties of LEDGF/p75 ΔPWWP/AT using the salt extraction assay (Figure 5a). LEDGF/p75 ΔPWWP/AT was fully extracted from chromatin at 100 mM NaCl in cells lacking HIV-1 integrase, as previously reported [13]. Interestingly, this extraction pattern was not modified in cells coexpressing LEDGF/p75 ΔPWWP/AT and HIV-1 integrase, indicating that the viral protein failed to promote chromatin binding of LEDGF/p75 ΔPWWP/AT.

In correlation with the results obtained with the salt extraction assay, LEDGF/p75 ΔPWWP/AT was exclusively recuperated in the non-chromatin bound fraction (S1) when cells were lysed in the presence of 150 mM NaCl (CSK I-150) (Figure 5b). This subcellular distribution of LEDGF/p75 ΔPWWP/AT was also observed in cells coexpressing HIV-1 integrase. Collectively, results in Figure 5 showed that HIV-1 integrase was unable to induce chromatin binding of LEDGF/p75 ΔPWWP/AT. Our results also correlate with a previous report indicating that the complex HIV-1 integrase-LEDGF/p75 ΔPWWP/AT does not associate with mitotic chromosomes [7].

### Effect of the ionic strength on the stability of the ternary complex chromatin LEDGF/p75-HIV-1 integrase

The HIV-1 co-factor activity of LEDGF/p75 involves its simultaneous interaction with the host chromatin and with HIV-1 integrase [1,2]. To determine the effect of the ionic strength on the interaction of HIV-1 integrase with chromatin bound LEDGF/p75, we evaluated the consequence of increasing concentrations of NaCl on the chromatin binding of HIV-1 integrase (salt extraction assay).

**Figure 6:**
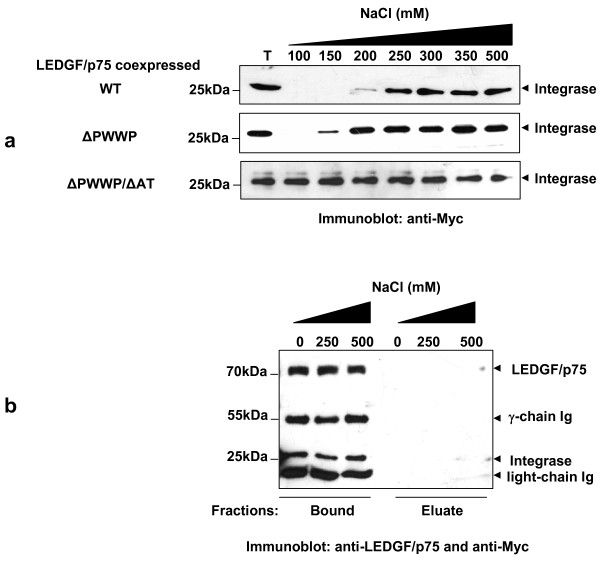
**Effect of the ionic strength on the stability of the ternary complex chromatin LEDGF/p75-HIV-1 integrase**. (a) The chromatin binding strength of the complex HIV-1 integrase-LEDGF/p75 was determined by the salt extraction method in LEDGF/p75-deficient HEK293T cells stably coexpressing Myc-tagged integrase and LEDGF/p75 WT, ΔPWWP or ΔPWWP/AT. Myc-integrase was determined by immunoblotting using an anti-Myc Mab. (b) Effect of salt concentration on the non-chromatin bound HIV-1 integrase-LEDGF/p75 complex. The integrase-LEDGF/p75 complex bound to anti-LEDGF Mab coupled-magnetic beads was incubated in CSK I buffer supplemented with different NaCl concentrations and LEDGF/p75 and integrase were determined by immunoblotting with anti-LEDGF and anti-Myc Mabs, respectively, in the immunocomplex-bound to the magnetic beads or in the eluated fractions.

HIV-1 integrase was fully extracted from chromatin at NaCl concentrations of 250 mM, 200 mM and 100 mM in cells coexpressing LEDGF/p75 WT, ΔPWWP and ΔPWWP/AT, respectively (Figure 6a). Interestingly, the extraction pattern of HIV-1 integrase was similar to the one observed for the coexpressed LEDGF/p75 proteins (Figure 2b and 5a). These observations suggest that HIV-1 integrase lacks chromatin interaction upon LEDGF/p75 chromatin detachment. Alternatively, the interactions of LEDGF/p75 with integrase and LEDGF/p75 with chromatin could be disrupted at a similar ionic strength. To evaluate these hypotheses, we studied the effect of NaCl concentration on the LEDGF/p75-HIV-1 integrase interaction using a non-chromatin bound complex.

The LEDGF/p75-HIV-1 integrase complex was released from the chromatin by DNase and (NH_4_)_2_SO_4 _treatment and isolated by immunoprecipitation with an anti-LEDGF/p75 Mab. The immunoprecipitated complex attached to the immunobeads was further incubated in CSK I buffer containing 0, 250 and 500 mM NaCl and the presence of LEDGF/p75 and Myc-tagged HIV-1 integrase in the eluate or bound to the immunobeads was evaluated by immunobloting. Results in Figure 6b indicated that the LEDGF/p75-HIV-1 integrase interaction is very stable, resisting the treatment with 500 mM NaCl. The effect of higher salt concentrations on the stability of this complex was not evaluated since at 500 mM NaCl the antibody-LEDGF/p75 interaction began to be disrupted. Data in figure 6 demonstrated that the chromatin-LEDGF/p75 interphase is sustained by weaker interactions than the LEDGF/p75-integrase in this ternary complex.

## Discussion

Chromatin binding is central in the role of LEDGF/p75 in HIV-1 DNA integration [1,2,7]. Biochemical and cellular biology evidences indicate that chromatin bound LEDGF/p75 tethers integrase to the host chromatin suggesting a cargo role for integrase during this process [22,25]. Our data have expanded the understanding of the interactions of the integrase-LEDGF/p75 complex with chromatin showing an active role of integrase in this association. We have shown evidences that the chromatin binding strength of LEDGF/p75 WT and the chromatin biding mutant LEDGF/p75 ΔPWWP is significatively enhanced upon direct interaction with HIV-1 integrase. Integrase also rescued the binding of the LEDGF/p75 ΔPWWP mutant to mitotic chromosomes. However, some degree of chromatin binding of LEDGF/p75 is required for this enhancing effect of integrase, since the viral protein was unable to induce chromatin binding of the LEDGF/p75 ΔPWWP/AT mutant that does not interact with chromatin [8,12]. These observations indicate that LEDGF/p75 and integrase cooperate in their binding to chromatin.

Due to the central role of chromatin binding in the HIV-1 cofactor activity of LEDGF/p75, it is expected that the HIV-1 cofactor activity of LEDGF/p75 ΔPWWP mutant is impaired. However, conflicting results in regards to the relevance of the PWWP domain in the HIV-1 cofactor activity of LEDGF/p75 have been reported. While this domain was found to be essential for HIV-1 infection in LEDGF/p75-knockout mouse fibroblast [15], its was dispensable in LEDGF/p75-deficient human CD4+ T cells (this study and [7]). The enhancing effect of integrase on the chromatin binding strength of LEDGF/p75 could offer a molecular explanation for the conserved HIV-1 cofactor activity of the LEDGF/p75 ΔPWWP mutant observed in human cells. It is possible that the chromatin binding strength of LEDGF/p75 ΔPWWP is enhanced to levels that sustain HIV-1 cofactor activity upon binding to the integrase in the HIV-1 pre-integration complex. In support of this hypothesis, HIV-1 integrase failed to promote chromatin binding of LEDGF/p75 ΔPWWP/AT and this mutant exhibits very poor HIV-1 cofactor activity. These evidences indicate a direct correlation between the capability of HIV-1 integrase to promote chromatin binding of LEDGF/p75 mutants and their HIV-1 cofactor activity.

Integrase binds nonspecifically to DNA through its C-terminal domain although all three integrase domains interact with DNA (reviewed in [16,17]). The N-terminal domain is located in close proximity to the integration target DNA whereas the catalytic core and the C-terminal domains participate in binding to viral LTR DNA. *In vitro *binding of LEDGF/p75 to HIV-1 integrase significantly increases the affinity of the viral enzyme for DNA [18]. We speculate that the binding of integrase to LEDGF/p75 increases its affinity for DNA and subsequently integrase enhances the binding of the LEDGF/p75-integrase complex to chromatin. In support to our model, during the preparation of this manuscript it was reported that HIV-1 integrase significantly enhances the affinity of LEDGF/p75 for chromatin as evaluated by quantitative fluorescence microscopy techniques [26].

The impaired HIV-1 cofactor activity of LEDGF/p75 ΔPWWP observed in mouse [15] but not in human cells (this study and [7]) could be a consequence of the method employed to express the LEDGF/p75 PWWP mutants in these cells. The LEDGF/p75 *null *mouse fibroblasts expressing LEDGF/p75 PWWP mutants were generated by transient coexpression of the mutant proteins and eGFP followed by fluorescence-activated cell sorting of the transfected cells [15], while the LEDGF/p75-deficient human CD4+ T cells were engineered to stably express the LEDGF/p75 mutant from the MLV promoter ([7] and this report). Transient but not stable expression, usually leads to protein overexpression. Then, it is possible that the overexpressed LEDGF/p75 PWWP mutants accumulate in the cytoplasmic compartment of the transfected mouse fibroblasts as a result of the saturation of the nuclear import pathway responsible for the nuclear localization of these mutants [12]. Different from LEDGF/p75 WT that enters to the nucleus via the nuclear import machinery during interphase [12,27] and by interacting with condensed chromatin during mitosis [12], LEDGF/p75 ΔPWWP depends exclusively on the nuclear import pathway to gain nuclear access [12]. Therefore, LEDGF/p75 ΔPWWP overexpression could generate a pool of cytoplasmic LEDGF/p75 able to sequester incoming viral pre-integration complexes in the HIV-1 challenged cells, impairing in this manner viral infection. In support of this mechanism, it has been demonstrated that other non-chromatin bound IBD-containing proteins interact with the integrase present in the incoming pre-integration complex in the cytoplasm compartment [28] blocking HIV-1 infection [1,7,7,28,29]. The generation of this LEDGF/p75 cytoplasmic pool is unlikely to occur in the absence of protein overexpression such as during LTR-driven stably expression of LEDGF/p75 PWWP mutants in the studied human cells. Variability in the levels of overexpressed LEDGF/p75 PWWP mutants in the transiently transfected mouse fibroblasts could also explain the paradoxical observation that some LEDGF/p75 PWWP domain point mutants have a significantly lower HIV-1 cofactor activity than mutants lacking the entire PWWP domain [15].

The formation and stability of the ternary complex chromatin LEDGF/p75-integrase are fundamental for HIV-1 infection [1-3]. Based on the effect of the ionic strength on the stability of this complex, we conclude that LEDGF/p75 establishes stronger interactions with HIV-1 integrase than with chromatin. In addition to binding to lentiviral integrases, LEDGF/p75 interacts through IBD with cellular proteins including the Myc-interactor protein JPO2 [30,31], the domesticated transposase of the pogo transposable element with ZNF domain (pogZ) [32], the menin/MLL histone methyltransferase complex [4] and the S-phase kinase Cdc7:ASK heterodimer [33]. The surface of interaction of the IBD with a subset of these proteins partially overlaps with that of integrase (reviewed in [19]). The affinity of IBD for integrase is higher than for some of these cellular proteins, explaining why they fail to restrict HIV-1 infection. The affinity of the catalytic core domain of HIV-1 integrase for recombinant LEDGF/p75 was approximately two-fold higher than that of pogZ or JPO2, as calculated in an AlphaScreen interaction assay [32]. Importantly, our data using LEDGF/p75-integrase complex immunopurified from cells corroborate these *in vitro *observations. We observed that the LEDGF/p75-integrase complex was stable in the presence of up to 500 mM NaCl whereas 400 mM NaCl was reported to significantly disrupt the interaction of LEDGF/p75 with pogZ [32]. This evidence suggests that HIV-1 integrase has evolved a high affinity surface of interaction with IBD that allows the viral protein to out compete the binding of other cellular IBD interactors.

In summary, our data indicate that HIV-1 integrase modulates the chromatin binding strength of the integrase-LEDGF/p75 complex and we propose that this effect of integrase determines the nonessential role of the PWWP domain of LEDGF/p75 in its HIV-1 cofactor activity.

## Conclusions

The new model that emerges from our study indicates that HIV-1 integrase enhances LEDGF/p75 chromatin binding during HIV-1 DNA integration and that this effect has important functional implications for the HIV-1 cofactor activity of LEDGF/p75.

## Methods

### Plasmids

#### LEDGF/p75 expression plasmids

pLEDGF/p75-IRES-Zeocin was used for stable expression of LEDGF/p75 WT or mutants in LEDGF/p75-deficient HEK293T cells (si1340/1428 cell line) [22]. This plasmid encodes a LEDGF/p75 cDNA linked to the gene conferring resistance to Zeocin. The re-expressed LEDGF/p75 contains seven synonymous mutations in the target site of the twenty-one shRNA 1340 [22]. This shRNA is present in all the LEDGF/p75-deficient cell lines used in this report. LEDGF/p75 mutants were generated on pLEDGF/p75-IRES-Zeocin with the Phusion™ Site-Directed Mutagenesis Kit (Finnzymes, Inc), as previously described [13]. The sequences of the specific primers used are available upon request. All the constructs described in this study were verified by overlapping DNA sequencing of the complete LEDGF/p75 cDNA.

#### HIV-1 integrase expression plasmids

pHIN [22] and pHIN-eGFP-IRES-P[13] are CMV-driven expression plasmids that encode HIV-1 integrase C-terminally tagged with Myc or Myc-eGFP, respectively.

Retroviral vector plasmids. Plasmids encoding single-round infection HIV-1 luciferase reporter vector (pHIVluc [1]), LEDGF/p75-expression MLV-derived vector (pJZLEDGF/p75 ΔPWWP [13]), and the Vesicular Stomatitis Virus glycoprotein G (VSV-G) expression plasmid pMD.G (a gift of D. Trono) were used for production of retroviral vectors.

### Cell lines

The human LEDGF/p75-deficient CD4+ T cell line, T_L3 _[1], was used for stable expression of LEDGF/p75 mutants while the T_C3 _cells expressing normal levels of LEDGF/p75 were used as control in some experiments. These cells were generated by transduction of SupT1 cells with an HIV-derived vector expressing a shRNA against LEDGF/p75 (T_L3_) or a control shRNA (T_C3_). T_L3 _cells express 97% less LEDGF/p75 mRNA than T_C3 _cells as determined by real time PCR [1]. T_L3 _LEDGF/p75 ΔPWWP cells were generated by transduction with an MLV-derived viral vector (pJZ308) harboring an expression cassette for this mutant and further selection in the presence of G418, as previously described [1]. The development and characterization of T_L3 _LEDGF/p75 WT cells was described before [13].

The LEDGF/p75-deficient HEK293T-derived cell line si1340/1428 [22] and the LEDGF/p75-deficient HEK293T cells expressing Myc-tagged HIV-1 integrase [13] were used for stable expression of the LEDGF/p75 WT or the deletion mutants ΔPWWP and ΔPWWP/AT. For generation of these stable cell lines, cells were plated at a density of 3 × 10^6 ^in a 75-cm^2 ^flask and transfected by calcium-phosphate the next day with 20 μg of the corresponding expression plasmids pLEDGF/p75-IRES-Zeocin linearized at the prokaryotic backbone. Stably transfected cells were obtained after selection in the presence of zeocin (150 ug/ml) and the expression of LEDGF/p75 WT and mutants was verified by immunoblotting with an anti-LEDGF/p75 Mab, as described below.

SupT1-derived cell lines were grown in RPMI1640 while HEK293T-derived cells were grown in DMEM and both culture media were supplemented with 10% heat-inactivated fetal calf serum, 2 mM L-glutamine and 1% penicillin/streptomycin.

### Real-time PCR quantification of LEDGF/p75 mRNA levels

The level of endogenous mRNA LEDGF/p75 was quantified in T_C3_, T_L3 _and T_L3 _cells expressing different LEDGF/p75 proteins. Primers sense MB36 (5' AGATGAACTTCAGGGTCAGC 3') and antisense MB60 (5' GTTTTATTCGCTTCCTCATGCTGTCT 3') were used for detection of endogenous LEDGF/p75. Primer MB60 binds to a nucleotide sequence that is present in the endogenous LEDGF/p75 mRNA but not in the MLV-expressed LEDGF/p75 mRNA. This sequence was targeted for mutagenesis in the MLV-expressed LEDGF/p75 cDNA to prevent recognition of the encoded mRNA by the LEDGF/p75-specific shRNA present in T_L3 _cells. As a result, seven synonymous mutations were introduced in this cDNA sequence avoiding binding of MB60. Primer MB36 binds to a region in LEDGF/p75 that is present in both the endogenous and the MLV-expressed LEDGF/p75 mRNAs. GAPDH mRNA levels were quantified using primers sense JK9 (5' CCCCTCCTAGGCCTTTGC 3') and antisense JK10 (5' GCTGAGAGGCGGGAAAGTT 3'). Total RNA was extracted from 4 × 10^6 ^cells using the RNeasy mini kit (Qiagen # 74104) and cDNA was generated from 2 μg of RNA with random oligonucleotides using the High Capacity cDNA Reverse Transcription Kit (Applied Biosystems #4368814). Quantitative real-time PCR was conducted with 12.5 ηg cDNA and 10 pM of the forward and reverse primers. Primers were annealed at 64°C for 30 secs and extension was allowed for 30 secs at 72°C, 50 PCR cycles were performed. Ct values for LEDGF/p75 and GAPDH were calculated comparing the results obtained with each sample to results obtained with a standard curve generated from a serial dilution of human genomic DNA (1ηg, 0.1ηg and 0.01 ηg; R^2 < 0.996) that was subject to PCR amplification with the primers described above. Next, the LEDGF/p75 mRNA levels were normalized to GAPDH mRNA levels and the fold change was calculated for endogenous LEDGF/p75 in control cells (T_C3_) vs T_L3_-derived cell lines.

### Generation of retroviral vectors

Procedures previously described were followed for the production of the single-round infection HIV-1 reporter virus and MLV-derived vectors [1]. Briefly, HIV-reporter virus (HIVluc) was prepared by calcium-phosphate cotransfection of HEK293T cells with 15 μg of pHIVluc and 5 μg of pMD.G. MLV-derived vectors were produced in Phoenix A packaging cells by calcium-phosphate cotransfection of 15 μg of pJZLEDGF/p75 ΔPWWP and 5 μg of, pMD.G. Forty-eight hrs after transfection, the viral supernatants were harvested. MLV-derived vectors were further concentrated by ultracentrifugation at 124,750 g for two hrs on a 20% sucrose cushion. Viral preparations were stored at -80°C until use.

### Single-round viral infectivity assay

T_L3 _and LEDGF/p75-expressing T_L3 _cells were plated at 1 × 10^5 ^cells in 500 μl of RPMI 1640 culture medium in 24-well plates and infected with HIVluc viral supernatants. Five days post-infection, cells were collected by centrifugation at 1,000 × g for six mins and the pellet lysed in 100 μl of PBS-1% Tween 20 for 15 mins on ice. Cellular lysates were centrifuged at 22,000 × g for 2 mins and supernatant used for quantification of luciferase activity. An aliquot of 20 μl of the cellular lysate supernatant was mixed with 45 μl of substrate (Bright-Glow™ Luciferase Assay System, Promega) and luciferase activity was quantified using a microplate luminometer.

### Immunoblotting

Cellular lysates were resolved by SDS-PAGE and transferred overnight to PDVF membranes at 100 mAmp at 4°C. Membranes were blocked in TBS containing 10% milk for one hour and then incubated in the corresponding primary antibody diluted in TBS-5% milk-0.05% Tween 20 (antibody dilution buffer). FLAG-tagged LEDGF/p75 was detected with anti-FLAG Mab (1/500, M2, Sigma) while non-tagged LEDGF/p75 was detected with an anti-LEDGF/p75 (1/500, clone 26/LEDGF, BD Biosciences), Myc-tagged HIV-1 integrase was detected with anti-Myc Mab (1/500, clone 9E10, Covance) and alpha tubulin was detected as a loading control with anti-alpha tubulin Mab (1/4000, Clone B-5-1-2 Sigma). Membranes were incubated overnight at 4°C with anti-FLAG, -LEDGF, or -Myc Mabs whereas anti-alpha tubulin Mab was incubated for two hrs at 25°C. Primary antibody-bound membranes were washed in TBS-0.1% Tween 20 and bound antibodies detected with goat anti-mouse Igs-HRP (1/2000, Sigma) followed by chemoluminescence detection. Densitometry analysis of immunoblots was performed with the gel analysis software UN-SCAN-IT gel 6.1 (Silkscientific).

### Transient expression of LEDGF/p75 and HIV-1 integrase in LEDGF/p75-deficient cells

LEDGF/p75-deficient 293T cells (si1340/1428 cells) [22] were plated at 0.45 × 10^6 ^cells/well in a six-well plate and transfected by calcium-phosphate with 1 ug of FLAG-tagged LEDGF/p75 and/or Myc-eGFP-tagged HIV-1 integrase expression plasmids. Transfected cells showing similar eGFP levels 48 hrs after transfection were analyzed using the salt extraction assay [13] as described below.

### Chromatin binding assay

Previously described procedures were followed [13] with minor modifications. Briefly, cells expressing different LEDGF/p75 mutants were lysed in CSK I or a modified CSK I buffer that contains 150 mM NaCl and fractionated by centrifugation to obtain a supernatant containing non-chromatin bound proteins (S1) and a pellet (P1) that represents the chromatin-bound fraction. Chromatin-bound proteins in P1 were further extracted from chromatin by treatment with DNase followed by incubation in (NH_4_)_2_SO_4 _250 mM (S2 fraction), while the insoluble non-chromatin bound proteins were retained in the pellet (P2 fraction). A total fraction (T) was obtained by lysing the cells in 100 μl of Laemmli buffer.

### Salt extraction assay

Procedures described in [13] were followed. Briefly, cells were lysed in CSK I buffer supplemented with increasing concentrations of NaCl. Cell cultures at 90% confluence were used to enrich for cells in interphase. Cellular lysates were then fractionated by centrifugation to soluble and insoluble fractions. The presence of LEDGF/p75 and/or HIV-1 integrase was determined in the soluble fraction by immunobloting. A total fraction was obtained by lysing the cells in Laemmli buffer.

### Integrase to chromatin tethering assay

This assay was previously described [13] and is based on the capacity of LEDGF/p75 to tether HIV-1 integrase to chromatin [22]. 2L_KD_-IN-eGFP cells that lack LEDGF/p75 and stably express HIV-1 integrase-eGFP were used in this assay. Briefly, cells were plated at 2 × 10^5 ^cells/chamber in 2 mls in a LabTek II chambered coverglass and transiently transfected the next day with LEDGF/p75 WT or mutants expression plasmids using the calcium phosphate method. Forty-eight hrs later, cells were fixed with 4% formaldehyde-PBS, stained with DAPI and used for analysis of the subcellular distribution of HIV-1 IN-eGFP-LEDGF/p75 complex by fluorescence microscopy.

### Immunofluorescence

2L_KD_-IN-eGFP cells were transfected and fixed with 4% formaldehyde-PBS as described above. Fixed cells were incubated with anti-LEDGF Mab (clone 26/LEDGF, BD Biosciences, 1/1,000 dilution) for 2 hrs at 37°C and bound antibodies were detected by incubation with anti-mouse Ig (H+L) coupled to Alexa Fluor 594 (10 μg/ml, Invitrogen A21203) for 45 mins at 37°C. Finally, the slides were stained with DAPI. The subcellular distribution of LEDGF/p75 and HIV-1 integrase proteins was analyzed by fluorescence microscopy.

### Sequencing of the MLV-transduced LEDGF/p75 cDNA stably expressed in T_L3_-derived cell lines

T_L3 _cells were engineered to express LEDGF/p75 WT and mutants by transduction with an MLV-derived viral vector as previously described [1]. For sequencing the MLV-delivered LEDGF/p75 cDNA, genomic DNA was extracted (High pure PCR template preparation kit, Roche) from 10^6 ^cells and 20 ηg of DNA was used for PCR amplification with Phusion™ DNA polymerase (Finnzymes, Inc). Primers sense MB66 (5' TCAACGGGACTTTCCAAAATGTCG 3') and antisense MB67 (5' AGATGAACTTCAGGGTCAGC 3') were annealed at 62°C and polymerase extension was allowed for 1.5 mins at 72°C, forty PCR cycles were performed. Next, the PCR product was resolved on an agarose gel electrophoresis and a DNA band of 1.3 Kb was isolated and sequenced.

### Analysis of the stability of the LEDGF/p75-HIV-1 integrase complex

HEK293T-derived LEDGF/p75-deficient cells stably expressing LEDGF/p75 and Myc-tagged HIV-1 integrase were treated as described in the chromatin binding assay to obtain the S2 fraction [13]. The S2 fraction corresponding to 6 × 10^6 ^cells was then incubated for 3 hrs at 4°C with 300 μl of goat anti-mouse Igs-coated magnetic beads (Pierce) that were previously loaded with 2 μg of anti-LEDGF Mab (BD Transduction Laboratories, catalog number 611714). Then, magnetic beads were washed four times with CSK I buffer supplemented with protease inhibitors (final concentration: leupeptine 2 μg/ml, aprotinin 5 μg/μl, PMSF 1 mM, pepstatin A 1 μg/ml) and further incubated for 15 mins on ice with

CSK I buffer containing protease inhibitors and different concentrations of NaCl.

Immunobeads-bound and non-bound (eluate) fractions were obtained by magnetic separation and analyzed by immunoblotting for LEDGF/p75 and Myc-integrase with anti-LEDGF and anti-Myc Mabs, respectively.

## Abbreviations

LEDGF/p75: Lens epithelium-derived growth factor/p75; IBD: integrase-binding domain; AT: AT hook motif; HIVluc: single-round infection HIV-1 luciferase reporter virus; MLV: murine leukemia virus.

## Competing interests

The authors declare that they have no competing interests.

## Authors' contributions

PA, EM, MTDB, JRK and JAGR performed the experiments, analyzed the results, and wrote the manuscript. ML planned the experiments, analyzed the results, and wrote the manuscript. All authors read and approved the final manuscript.
